# Design, Manufacture and Test of Piezoelectric Cantilever-Beam Energy Harvesters with Hollow Structures

**DOI:** 10.3390/mi12091090

**Published:** 2021-09-10

**Authors:** Baozhi Wang, Chenggong Zhang, Liyan Lai, Xuan Dong, Yigui Li

**Affiliations:** 1 School of Electrical and Electronic Engineering, Shanghai Institute of Technology, Shanghai 201418, China; bzwang125@outlook.com; 2 School of Science, Shanghai Institute of Technology, Shanghai 201418, China; zcg1006@126.com (C.Z.); lylai@sit.edu.cn (L.L.); dx18521076578@163.com (X.D.)

**Keywords:** bulk PZT, low frequency, high power density, hollow out, single-crystal PEH

## Abstract

This article presents a single-crystal piezoelectric energy harvester (PEH) with a trapezoidal hollow hole that can obtain high energy density at low frequency. Harvesters with a hollow structure were fabricated through a series of manufacturing processes such as thermocompression bonding, screen printing and laser cutting. Finite element analysis (FEA) and experimental results showed that using low modulus brass instead of stainless steel as the PEH substrate enhances the voltage output of the device, and the hollow design greatly increases the overall stress level and power density. In addition, the developed PEH with a trapezoidal hole obtained the best output performance; when the acceleration, resonance frequency and matched load resistance were 0.5 g, 56.3 Hz and 114 kΩ, respectively, the peak voltage was 17 V and the power density was 2.52 mW/cm^3^. Meanwhile, compared with the unhollowed device, the peak voltage and maximum power density of the proposed PEH were increased by 30.7% and 24.4%, respectively, and the resonance frequency was reduced by 7%. This study verified the feasibility of the optimized design through simulation and experimental comparison.

## 1. Introduction

With the development of vibration energy harvesting technology, piezoelectric materials are playing an increasingly important role in the field of self-power supply [1,2,3], and piezoelectric energy harvesters (PEHs) are gradually being used in various smart sensors and wireless network nodes or systems [4,5,6]. Piezoelectric materials mainly include piezoelectric single crystals, ceramics, polymers and composite materials [7,8], while bulk PZT in piezoelectric ceramics has been widely studied due to its high-voltage electrical constant and electromechanical coupling coefficient [9]. Meanwhile, harvesters designed with bulk PZT have the unique advantages of simple structure, high energy density and high conversion efficiency. Therefore, they can be used to collect and supply energy from various vibration sources in the environment [10,11,12,13]. Currently, there are three main types of reported applications: assembled PEHs, which are mainly applied in road and vehicle suspension [14,15,16], wearable PEHs, which are usually used as an accessory on human body surfaces such as the arm and neck [17,18], and implantable PEHs, such as pacemakers and cochlear implants [18,19,20,21].

However, piezoelectric energy harvesting technology still faces a number of challenges. Harvesters with low frequency, high power density and good stability are urgently needed to meet the requirements of energy development [3,7]. In recent years, the main challenge is that the device needs to match the lower resonance frequency of the vibration source. Typically, the resonant frequency can be reduced by increasing the proof mass of the PEH or thinning the piezoelectric layer, but this increases its overall size and reduces the energy output. Eventually, this leads to problems such as the need for a large space to ensure system integration and insufficient energy supply. In addition, conventional silicon substrates are extremely vulnerable to damage due to their high modulus characteristics [22]. Therefore, material optimization, substrate replacement and geometry change are the main methods currently utilized for the optimization of harvesters [23,24,25,26,27]. Dong et al. [23] proposed a dual-crystal PEH that was asymmetric from top to bottom and studied the effect of different coverage PZT lengths on device performance. The device obtained a 26.9 mW/cm^3^ maximum power density at 1.5 g. In order to obtain a large amount of stress/strain from the clamping end, Nabavi and Zhang [24] proposed a new T-type MEMS piezoelectric energy harvester with high power density and low resonant frequency. Compared with the traditional cantilever-beam device, the T-type PEH increased the energy conversion efficiency by 4.8 times. To address the problem of the low energy density of PEHs, Hajheidari et al. [25] studied a non-uniform bimorph piezoelectric chip with different parameter designs, and verified the accuracy of the experiment through numerical analysis and theory. In terms of geometric optimization, Nisanth et al. [26] studied the performance of PEHs under different mass shapes. Compared with the PEH array with the traditional rectangular mass, the PEH array with the triangular mass increased the power output by 1.79 times at 0.5 g. Jin et al. [27] studied the influence of different width beam shapes and electrode arrangements on the voltage output of harvesters, and verified through simulation and experimentation that the parabolic shape beam resulted in the best improvement in the performance of the devices. Differently shaped PEHs make the preparation and bonding of the proof masses difficult; however, the extension of linear PEHs will expand the options for device integration.

In this paper, a high-performance cantilever beam-based PEH with a trapezoidal hollow structure was proposed. The stress level and charge density of traditional and hollowed-out PEHs were obtained by finite element analysis. The influence of the hollow structures on the stress distribution and charge density of harvesters was analyzed in the resonance state. The designed hollow harvesters realized the improvement and expansion of the stress distribution of the piezoelectric layer. The fabricated trapezoidal hollow PEH obtained the best power density at a lower resonance frequency, which makes the application of the device in WSNs more flexible.

## 2. Design and Simulation

**Figure 1:**
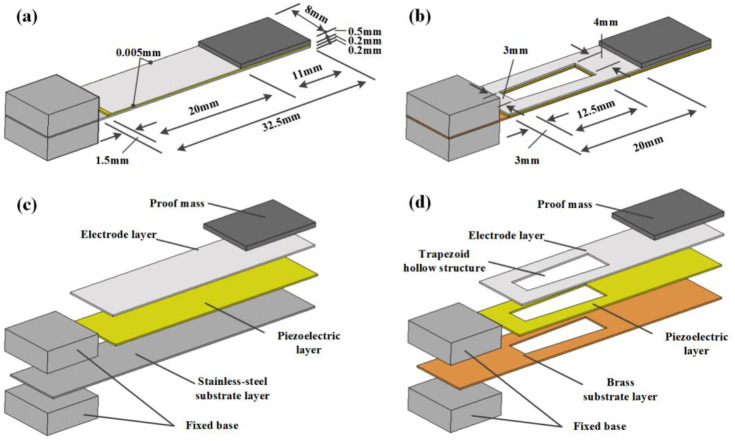
Schematic diagram of piezoelectric energy harvesters. (**a**) The traditional structure of a PEH with stainless steel substrate. (**b**) The proposed hollow structure of a PEH with brass substrate. (**c**) Components of a traditional PEH. (**d**) Components of the proposed PEH.

Figure 1 shows the overall size of the design and the structural components of the piezoelectric energy harvesters. The left end is the fixed end of the cantilever, which is fastened to the excitation platform on fixed bases; the right end is the free end of the cantilever, and the proof mass is glued to the top (Figure 1a). The elastic layer of the harvesters includes an electrode layer, a piezoelectric layer and a substrate layer (Figure 1c). Figure 1b presents the high-performance PEH proposed in this article. It is based on a traditional PEH, but the stainless steel substrate was replaced with brass and the elastic layer structure was hollowed out (Figure 1d).

The materials and parameters used for the PEHs in the simulation and experiment are shown in Table 1. The piezoelectric coefficient matrix, elastic coefficient matrix and dielectric coefficient matrix of PZT-7 are shown in Formula (1), Formula (2) and Formula (3), respectively. The stainless steel-based PEH (S-PEH) and the brass-based PEH (B-PEH) with the traditional structure were studied separately. The electrode layer was ignored during the simulation.

(1) $$d=\left\{\begin{array}{ccc} 0 & 0 & -207\\ 0 & 0 & -207\\ 0 & 0 & 410\\ 0 & 550 & 0\\ 550 & 0 & 0\\ 0 & 0 & 0 \end{array}\right.e^{-12}m/V$$

(2) $$s=\left\{\begin{array}{cccccc} 15.8 & -5.7 & -7.0 & 0 & 0 & 0\\ -5.7 & 12.7 & -7.0 & 0 & 0 & 0\\ -7.0 & -7.0 & 18.1 & 0 & 0 & 0\\ 0 & 0 & 0 & 40.6 & 0 & 0\\ 0 & 0 & 0 & 0 & 40.6 & 0\\ 0 & 0 & 0 & 0 & 0 & 43.0 \end{array}\right.e^{-12}m^{2}/N$$

(3) $$\varepsilon_{r}=\left\{\begin{array}{ccc} 1930 & 0 & 0\\ 0 & 1930 & 0\\ 0 & 0 & 2100 \end{array}\right.$$

**Figure 2:**
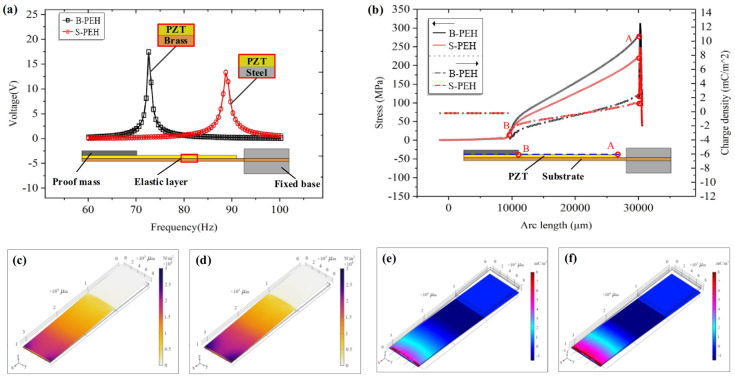
(**a**) Voltage output of S-PEH and B-PEH at different frequencies. (**b**) The stress level and charge density on the outside of the piezoelectric layer. (**c**) Surface stress distribution of S-PEH. (**d**) Surface stress distribution of B-PEH. (**e**) Surface charge density of S-PEH. (**f**) Surface charge density of B-PEH.

The coupling analysis of solid mechanics, electrostatic and piezoelectric effects on PEHs was performed by Comsol Multiphysics software. Free tetrahedrons are used for meshing. The total number of units was 89,012, the damping of the linear elastic material was set as 0.001, and the isotropic loss factor was set as 0.0015. The voltage output of S-PEH and B-PEH at different frequencies was calculated through a frequency sweep. The frequency range was from 60 Hz to 100 Hz, and the step length was 0.5 Hz. The resonance frequency and peak voltage of B-PEH and S-PEH were 88.5 Hz, 13.5 V and 72.5 Hz, 17.7 V, respectively (Figure 2a). Figure 2b shows the simulated stress level and charge density on the outside of the piezoelectric layer (blue dotted line). The charge density and stress level are concentrated at position A near the fixed end, and continue to decrease toward the free end of the cantilever until it drops to zero at position B. The surface stress distribution and surface charge density of S-PEH and B-PEH at resonance frequency were plotted through post-processing. Under the same color scale, the maximum stress and charge density of B-PEH are higher than those of S-PEH.

According to the FEA results, the use of low modulus brass as the PEH substrate effectively increases the peak voltage by 31% compared with stainless steel-based PEH. By increasing the stress level of the elastic layer, the performance of the device can be successfully improved. According to the simulation results, PEHs with the above two substrates were prepared for comparative experimental testing.

## 3. Fabrication and Test Results of PEH

**Figure 3:**
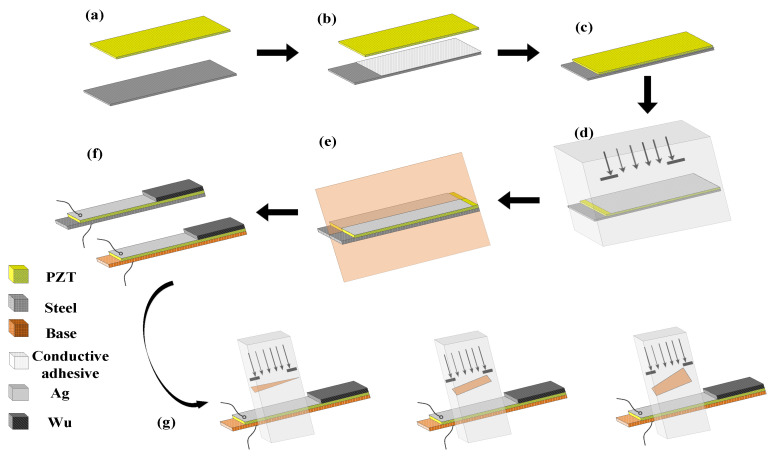
Manufacturing procedure for the PEH: (**a**) Polishing the piezoelectric ceramic and stainless steel. (**b**) Screen printing bonding layer. (**c**) Thermal compression bonding of bulk PZT and metal layer. (**d**) Screen printing of the upper electrode layer. (**e**) Laser cutting. (**f**) Bonding of leads and proof masses. (**g**) Formation of hollowed-out devices.

The detailed manufacturing processes are shown in Figure 3. The single-crystal harvester prototype was fabricated according to the material parameters shown in Figure 1, and a multilayer composite structure processing and preparation process was adopted. The preparation process uses S-PEH as an example.

**Figure 4:**
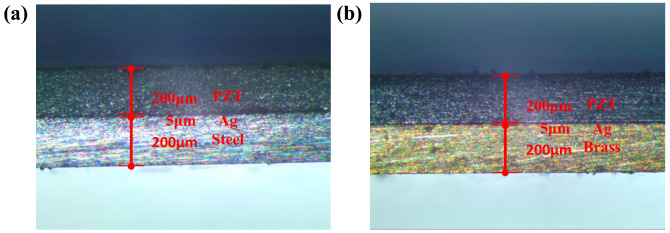
The cross-section (OM) of PEH. (**a**) Stainless steel substrate PEH. (**b**) Brass substrate PEH.

First, the designed PZT and stainless steel were ultrasonically cleaned for 10 min, placed in an oven at 80 °C to remove water; then, the polishing pad was coated with abrasive paste and then the bulk PZT and stainless steel were polished on both sides (Figure 3a). As shown in Figure 3b, a layer of about 5 μm of conductive silver glue was screen-printed on the surface of the stainless steel. Then the PZT was adhered to it by thermocompression bonding (Figure 3c), and the bonding conditions were kept at a constant temperature in a vacuum oven at 200 °C. After 3 h, the temperature was lowered until it dropped to room temperature. Since the thermal expansion coefficient of brass is higher than that of stainless steel, in order to prevent the metal layer from shrinking due to the sudden temperature drop, the temperature was lowered step-by-step during the preparation of the brass substrate device, and the temperature was kept constant for half an hour for every 30 °C change. Figure 4 illustrates the cross-section of the cantilever beam after bonding with images taken by a metallurgical microscope (DYJ-980BD, Shanghai, China). As shown in Figure 3d, a 5 μm-thick Ag electrode was screen-printed on to the upper surface of the PZT. In order to prevent the high-power laser (1064 nm, RZY-BX-10B, Suzhou, China) from causing PZT depolarization, the laser power was controlled at 5 W. The device was cut 2000 times with the power, scanning speed and frequency set to 5 W, 50 mm/s and 20 KHz, respectively (Figure 3e). The PEH prototype was completed with conductive silver glue bonding wire and a Wu mass block (Figure 3f). Finally, three types of hollowed out samples were formed by laser cutting under the above conditions for the upcoming tests (Figure 3g).

**Figure 5:**
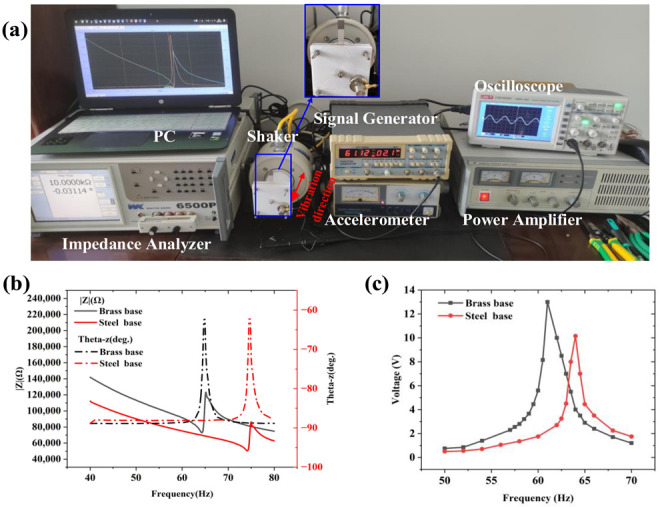
(**a**) Experiment and test platform of PEHs. (**b**) The impedance test results of the prepared S-PEH and B-PEH. (**c**) Voltage output of S-PEH and B-PEH at different frequencies.

The fabricated S-PEH and B-PEH were tested by an impedance analyzer as shown in Figure 5. The resonance frequency and matched loading resistance of the S-PEH and B-PEH were 64.3 Hz, 72.8 kΩ and 74.2 Hz, 50.3 kΩ, respectively. The vibration excitation system of the device is shown in Figure 5a, which includes a vibrating table (SINOCERA JZK-20, State College, PA, USA), a signal generator (UNI-T UTG9002C, Kowloon, HK), a power amplifier (SINOCERA YE5872A, State College, PA, USA), an oscilloscope (UNI-T UTD2102CEX, Kowloon, HK) and an acceleration monitor (SINOCERA YE5932, State College, PA, USA). The vibration direction of the vibrating table remains horizontal. Under 0.5 g (g = 9.795 m/s^2^) acceleration excitation conditions, the resonance frequency, peak voltage and maximum output power of S-PEH and B-PEH were obtained as 61 Hz, 10.15 V, 0.256 mW and 64 Hz, 13 V, 0.290 mW, respectively. The output power was calculated by the formula, $P_{eff}=V_{p-p}^{2}/(8*R_{esistance})$. Compared with S-PEH, the prepared B-PEH increased the peak voltage by 28% (Figure 5c).

The FEA and experimental results illustrated that using low modulus brass instead of a stainless steel layer as the PZT substrate resulted in higher output power at lower resonance frequency. The resonance frequency and peak voltage of the harvesters obtained by FEA and the experimental tests show a certain deviation. The main reasons for this error are the assembly accuracy in the experiment and the neglect of dielectric loss and polarization loss in the simulation. According to the simulation results, the effective piezoelectric layer (i.e., charge density, stress level) of the device was mostly concentrated on the fixed end, and an attempt was made to obtain a uniformly distributed stress level by hollowing out the B-PEHs. To this end, three graphical hollow designs were demonstrated, and FEA and experimental tests were carried out for each of these.

## 4. Optimize Test Results

**Figure 6:**
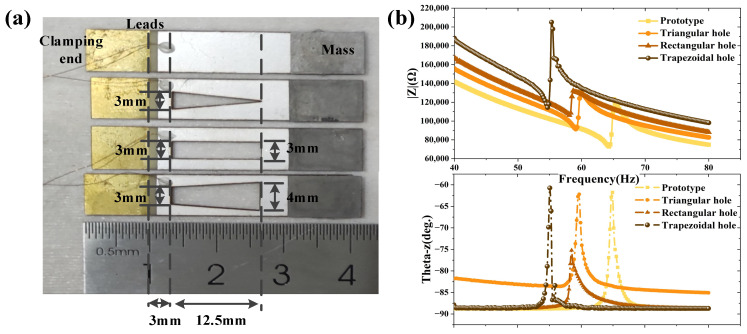
(**a**) The designed size of the hollow structures. (**b**) The impedance test results for different types of hollow holes.

For the purpose of controlling the variables, the proof masses of the four types of B-PEHs designed remain unchanged. Furthermore, in order to make the fixed ends of the piezoelectric material the same, the fixed ends (the upper and bottom of holes) were the same length. The fabricated harvesters and detailed design dimensions are shown in Figure 6a and Table 2. The best external loads for the unchanged, triangular hole, rectangular hole and trapezoidal hole devices, measured by the impedance analyzer were 72.8 kΩ, 91 kΩ, 106 kΩ and 114 kΩ, respectively (Figure 6b).

**Figure 7:**
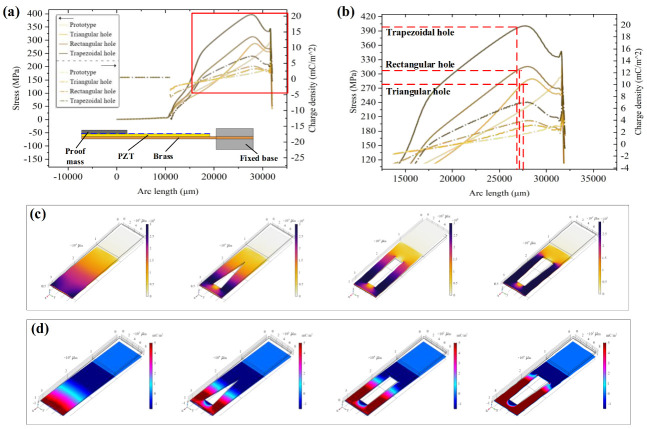
Simulation results of four types of piezoelectric energy harvesters. (**a**) The stress level and charge density on the outside of the piezoelectric layer. (**b**) Comparison of stress level and charge density of devices. (**c**) The surface stress levels of B-PEHs with different hollow structures. (**d**) The surface charge density of B-PEHs with different hollow structures.

As shown in Figure 7, the stress distribution and charge density of the devices at different resonant frequencies were obtained by FEA. Figure 7a presents a line graph of the stress level and charge density on the outside of the piezoelectric layer (the blue dotted line). It was found that the position of the maximum stress and the highest charge density of the devices with different hollow structures were consistent. The unhollowed B-PEH prototype obtains maximum stress at the root of the piezoelectric layer and the stress decreases toward the free end. The PEHs with the three hollow structures effectively increase the root stress and gradually increase the stress toward the free end. Once the maximum stress is obtained, the stress is gradually reduced to zero. On the whole, the stress level and charge density of the PEHs with hollow structures are higher than those of the unhollowed prototype. Among the three hollow structures, the B-PEH with a trapezoidal hole has the most obvious effect on the stress level and charge density expansion (Figure 7b). From the surface stress distribution and surface charge distribution shown in Figure 7c,d, it can be easily observed that under a uniform color scale, the stress level and charge density of harvesters with hollow structures continue to extend to the free end of cantilever, and the B-PEH with a trapezoidal hole has the widest stress and charge density distribution.

**Figure 8:**
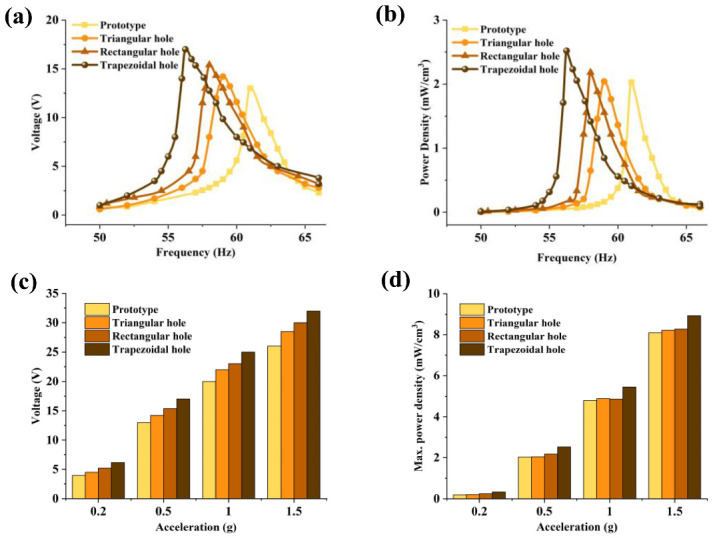
Output performance of various types of piezoelectric energy harvesters. (**a**) Voltage output of various devices at different frequencies. (**b**) Power density of various devices at different frequencies. (**c**) Maximum peak voltage of various devices at different accelerations. (**d**) Maximum power density of various devices at different accelerations.

When tests were conducted at an acceleration of 0.5 g, the peak voltage output and power density of the PEH with non-hollow, triangular, rectangular and trapezoidal holes were 13 V, 14.2 V, 15.4 V, 17 V and 2.026 mW/cm^3^, 2.041 mW/cm^3^, 2.182 mW/cm^3^, 2.520 mW/cm^3^, respectively (Figure 8a,b). Under resonant frequencies of 61 Hz, 59 Hz, 57.9 Hz and 56.3 Hz, respectively, the maximum voltage output and maximum power density of each device under applied accelerations of 0.2 g, 0.5 g, 1.0 g and 1.5 g are shown in Figure 8c,d. The peak voltage and maximum power density of the trapezoidal hole device were 32 V and 8.932 mW/cm^3^ at 1.5 g, respectively. Table 3 presents the comparison between the performance of various types of harvesters.

## 5. Conclusions

In summary, this article outlines the design, manufacture and test of cantilever-beam based PEHs with hollow structures. By replacing the stainless steel substrate layer with brass, the peak voltage and output power of the manufactured B-PEH was increased by 28% and 13%, respectively, compared with the S-PEH, and the resonance frequency was reduced by 5%. On this basis, B-PEHs with three types of hollow holes, including a triangular hole, a rectangular hole and a trapezoidal hole were fabricated by laser processing technology. The FEA and test results showed that the hollow design can effectively increase the stress level and charge density of PEHs in the resonance state, and the PEH with the trapezoidal hollow has the best performance, enabling the device to provide higher power density at a lower natural frequency. Compared with the unhollowed B-PEH, the PEH with a trapezoidal hole reduced the resonant frequency by 7% and increased the power density by 24.4%. Under 1.5 g of acceleration, the PEH had a power density of 8.932 mW/cm^3^.

## Figures and Tables

**Table 1:** Material properties of different structures.

Structure	Substrate Layer		Piezoelectric Layer	Mass	Electrode Layer
Material	Stainless steel	Brass	PZT-7	Wu	Ag
Young’s modulus (GPa)	200	112	-	411	83
Density (kg/m^3^)	7850	8960	7800	19,350	10,500
Poisson’s ratio	0.30	0.34	0.36	0.28	0.37

**Table 2:** Designed size of different types of hollow holes.

Types	Triangle Hole	Rectangular Hole	Trapezoidal Hole
Upper bottom (mm)	3	3	3
Lower bottom (mm)	0	3	4
Height (mm)	12.5	12.5	12.5
Hole area (mm^2^)	18.75	37.5	43.75

**Table 3:** Comparison of the performance of various types of harvesters.

Types of PEHs	Acceleration (g)	Resonant Frequency (Hz)	Effective Volume (mm^3^)	Optimal Load Resistance (kΩ)	Max. Power Density (mW/cm^3^)
Steel-based Prototype	0.5	64	143.2	50.3	1.788
Brass-based Prototype	0.5	61	143.2	72.8	2.026
Triangle hole	0.5	59	135.7	91	2.041
Rectangular hole	0.5	57.9	128.2	106	2.182
Trapezoidal hole	0.5	56.3	125.7	114	2.520
	1.5				8.932

## Data Availability

Data available on request due to restrictions, e.g., privacy or ethical. The data and material presented in this study are available on request from the corresponding author.
